# Incidence and prevalence of connective tissue disease (CTD)-associated interstitial lung disease in the United States

**DOI:** 10.1007/s10067-026-08480-6

**Published:** 2026-10-23

**Authors:** Diana Martins, George Mu, Elaine Irving, Sally Wetten, Roger A. Levy, Keele Wurst

**Affiliations:** 1 Global Epidemiology & RWE, GSK, Mississauga, ON Canada; 2 Real World Analytics, GSK, Collegeville, PA USA; 3 Clinical Research, GSK, Stevenage, UK; 4 Global Epidemiology & RWE, GSK, Stevenage, UK; 5 Global Medical Affairs, GSK, Collegeville, PA USA; 6 Global Epidemiology & RWE, GSK, Durham, NC USA

**Keywords:** Connective tissue disease, Epidemiology, Incidence, Interstitial lung disease, Prevalence

## Abstract

**Objectives:**

Interstitial lung disease (ILD) is a pulmonary manifestation of connective tissue diseases (CTDs). Limited data exist describing the epidemiology of CTD-ILD; therefore, this study aimed to estimate the incidence and prevalence of CTD-ILD in the US, overall and by CTD-ILD subtypes.

**Methods:**

Patients with CTD-ILD and rheumatoid arthritis (RA), systemic sclerosis (SSc), systemic lupus erythematosus (SLE), idiopathic inflammatory myopathy (IIM), mixed CTD (MCTD), and Sjögren’s disease (SjD) were identified from Optum^®^ de-identified Electronic Health Record data set (Optum^®^ EHR) and MarketScan^®^ Commercial and Medicare US databases between 2019 and 2024.

**Results:**

ILD was present in 4.3% (n = 21,366/496,690; Optum^®^ EHR) and 3.2% (n = 7521/234,468; MarketScan) of identified patients with CTD. This proportion was highest for those with SSc (Optum^®^ EHR: 20.3%; MarketScan: 21.1%). Most patients with CTD-ILD were female in both Optum^®^ EHR and MarketScan, with median ages 64 and 56 years, respectively; race was available only in Optum^®^ EHR, where 72.1% were categorized as Caucasian. Age- and sex-adjusted incidence rates were 7.6 and 10.0 per 100,000 person-years, with adjusted prevalence rates 46.7 and 43.4 per 100,000 persons. Rates varied by CTD subtype and were highest for RA-ILD and lowest for MCTD-ILD across databases. Crude rates of CTD-ILD regardless of subtype were higher among female patients, patients aged ≥ 65 years, and among patients of Black African ancestry.

**Conclusion:**

Consistent age- and sex-adjusted CTD-ILD incidence and prevalence estimates between two large, demographically distinct US databases provide contemporary population-level estimates of CTD-ILD frequency and distribution that can inform disease screening and patient care.

**Table:** 

**Key points** • *US CTD-ILD incidence estimated at 7.6 and 10.0/100,000 person-years and prevalence estimated at 46.7 and 43.4/100,000 persons.* • *Incidence and prevalence rates were highest in female patients, patients aged ≥ 65 years, and patients of Black African ancestry.* • *Incidence and prevalence rates varied between CTD subtypes and were highest for RA-ILD and lowest for MCTD-ILD.*

**Supplementary Information:**

The online version contains supplementary material available at https://doi.org/10.1007/s10067-026-08480-6.

## Introduction

Connective tissue diseases (CTDs; or systemic autoimmune rheumatic diseases [SARDs]) are a heterogeneous group of immune-mediated diseases that affect connective tissues. Interstitial lung disease (ILD) is a common pulmonary manifestation associated with CTDs such as rheumatoid arthritis (RA), systemic sclerosis (SSc), systemic lupus erythematosus (SLE), idiopathic inflammatory myopathy (IIM), mixed CTD (MCTD), and Sjögren’s disease (SjD) [1–4]. ILD is characterized by aberrant immunologic dysregulation leading to vascular injury and fibrosis of the lung parenchyma [1, 3, 5].

Symptoms associated with ILD, such as breathlessness, cough, and fatigue, are associated with impaired quality of life [6]. Patients with CTD-ILD have poorer survival outcomes compared with those who have CTD alone, and ILD is a leading cause of mortality in this population of patients, with 5-year survival rates among patients with ILD ranging between 73% and 85%, depending on CTD subtype [7, 8]. Predictors of ILD development, progression, and mortality vary across CTD subtypes [2, 3, 7, 9]. Progression of disease following diagnosis is variable, with factors such as smoking history and age indicating increased risk [9].

Lack of disease awareness and early testing, and asymptomatic or non-specific symptom presentation, complicate diagnosis and clinical management of CTD-ILDs [10–12]. Despite CTD-ILD being associated with a considerable patient and socioeconomic burden [13, 14], there are only a few approved treatments. Tocilizumab is approved in the US for use in patients with SSc-ILD [15–17], and nintedanib in patients with SSc-ILD and those with CTD-ILD with a progressive fibrosing phenotype [18, 19]. These limited treatment options highlight a substantial and ongoing unmet clinical need for better treatment strategies [9]. This is also reflected in the clinical practice guidelines recently developed by the American College of Rheumatology (ACR) and the European Respiratory Society/European Alliance of Associations for Rheumatology (ERS/EULAR) [11, 20], which are largely based on recommendations for the off-label use of immunosuppressants based on clinical experience.

Although ILD is a recognized complication across CTDs, epidemiological data on its prevalence and incidence remain limited. Most existing research has focused on the more common individual CTD subtypes separately, using different definitions and/or case-identification algorithms. Alongside a gap in knowledge of the less common subtypes, it is challenging to create an overall picture of CTD-ILD prevalence and incidence [9, 14, 21, 22]. Because ILD risk and progression differs among CTD subtypes, a better understanding of its epidemiology is essential to inform screening, diagnosis, and intervention strategies [9]. This study aimed to estimate the incidence and prevalence of CTD-ILD, overall and by CTD-ILD subtypes, using a validated ILD algorithm [23] applied to two large United States (US) databases.

### Methods

#### Study design

This study included data from two retrospective cohort analyses from two large US databases, Optum^®^ de-identified Electronic Health Record data set (Optum^®^ EHR) (GSK Study 223907) and MarketScan^®^ Commercial and Medicare databases (GSK Study 224023) to enable broader population coverage and assess reproducibility. This study complied with all applicable laws regarding patient privacy and was conducted in accordance with the Declaration of Helsinki 2008 and its later amendments.

The Optum^®^ EHR database includes EHRs from a variety of hospital-based and ambulatory-only facilities across the US, capturing over 115 million individuals, 150,000 providers, 2000 hospitals, and 7000 clinics. Optum^®^ EHR includes demographic data and clinical characteristics such as laboratory results, medications, procedures, diagnoses, and hospitalizations. The MarketScan database is a collection of health insurance claims for working adults and retirees with employer-sponsored health insurance, but with a limited number of Medicare Advantage–insured patients, as the proportion of patients aged ≥ 65 is much lower than in US census reports. This commercial database contains all paid claims generated by millions of commercially insured individuals annually, with member identification codes that allow individuals to be followed longitudinally and standard codes for diagnoses, procedures, and drugs.

This study included adults aged ≥ 18 years with RA-ILD, SSc-ILD, SLE-ILD, IIM-ILD, MCTD-ILD, or SjD-ILD from the Optum^®^ EHR and MarketScan databases. CTD diagnoses were identified using International Classification of Diseases, 10th Revision (ICD-10) codes between January 1, 2019, and December 31, 2024 (Supplementary Table S1). ILD diagnosis was identified within 365 days before, on, or after the CTD diagnosis; this permitted ascertainment of CTD-ILD cases between 2019 and 2024. In the absence of a validated algorithm for CTD-ILD for individual subtypes, a validated algorithm for RA-ILD was used consistently across all subtypes to minimize variation [14]. The index date was defined as the later of the CTD and ILD diagnosis dates that met inclusion criteria. To identify incident cases, a 1-year washout period was used; only those who had at least 1 year of enrollment prior to index date and no ILD diagnosis code in that year were included as an incident case. All cases were required to have at least 1 day of enrollment during the study period (using a proxy definition in Optum^®^ EHR), be treated within an integrated delivery network, be aged ≥ 18 years at index date, and not have a death date prior to the index date.

Three sensitivity analyses were conducted. The first sensitivity analysis used a broader list of ICD-10 codes associated with ILD than the primary analysis, including disease-specific lung involvement (Supplementary Table S1). The second sensitivity analysis retained the primary analysis definitions but required at least 365 days of enrollment during the study period (vs ≥ 1 day in the primary analysis) to assess the effect of the enrollment criterion. In the third sensitivity analysis, patients with more than one CTD diagnosis were assigned to a separate multi-CTD-ILD group to assess the effect of overlapping CTD diagnoses on incidence and prevalence estimates.

#### Outcomes

Outcomes were the incidence (per 100,000 person-years [PY]) and prevalence (per 100,000 persons) rates of CTD-ILD overall and by CTD subtype between 2019 and 2024. For both incidence and prevalence, crude estimates were presented for CTD overall, by CTD subtype, and by sex, age, and race (only Optum^®^ EHR) within each database. Additionally, age- and sex-adjusted incidence and prevalence rates were reported for CTD overall and by CTD-subtype for each database.

#### Statistical analysis

The overall incidence rate was calculated as the number of new (incident) cases in the study period/total time at risk in the base population (PY × 100,000). Time at risk was calculated as first day of enrollment until the earliest of case definition date, end of enrollment, death, or end of study period. The overall prevalence rate was calculated as the number of cases/total number of patients with at least one day of enrollment within the study period in the base population × 100,000. For age- and sex-adjusted prevalence or incidence, adjustments were made using the US Census Population from the US Census Bureau by age and sex (Supplementary Methods). In all analyses except the third sensitivity analysis, identified patients could have more than one CTD diagnosis during the study period and were counted in more than one subtype. However, when reporting the CTD-overall group, patients were only counted once. Demographic characteristics of patients were described as of their index date.

## Results

### Population characteristics

**Fig. 1:**
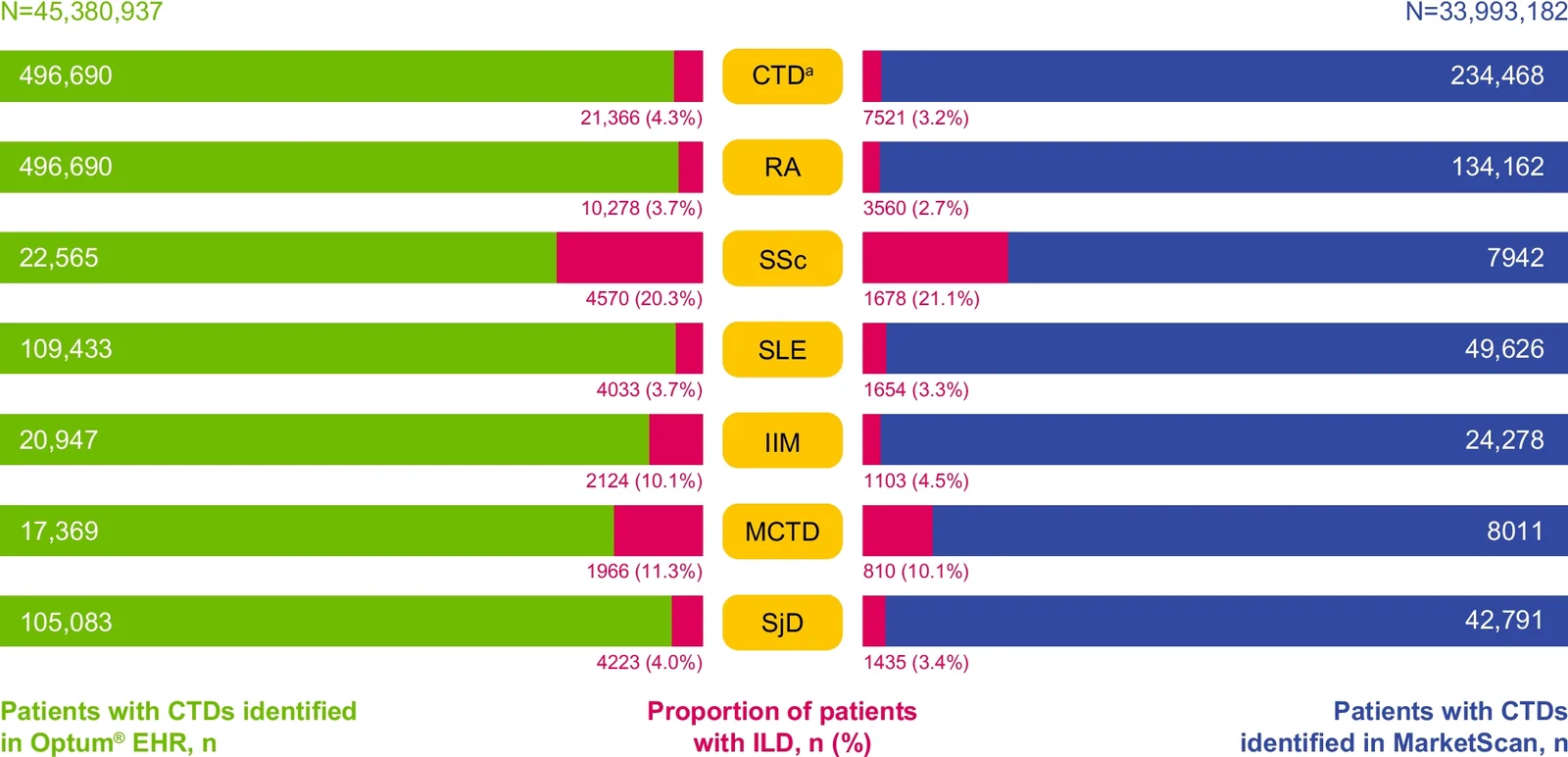
Proportion of patients with CTD^a^ and CTD-ILD identified in Optum^®^ EHR and MarketScan databases. ^a^Overall captures any CTD-ILD subtype (no duplicates), but patients may be captured in more than one CTD subtype. CTD, connective tissue disease; EHR, electronic health record; IIM, idiopathic inflammatory myopathy; ILD, interstitial lung disease; MCTD, mixed connective tissue disease; RA, rheumatoid arthritis; SjD, Sjögren’s disease; SLE, systemic lupus erythematosus; SSc, systemic sclerosis

From 45,380,937 (Optum^®^ EHR) and 33,993,182 (MarketScan) eligible individuals (2019–2024), 496,690 patients (Optum^®^ EHR) and 234,468 patients (MarketScan) with CTD were identified (Fig. 1). Among patients with all CTDs, those identified with the highest proportion of ILD were SSc (20.3%–21.1%) and MCTD (10.1%–11.3%), compared with other CTD subtypes: IIM (4.5%–10.1%), SjD (3.4%–4.0%), SLE (3.3%–3.7%) and RA (2.7%–3.7%) (Fig. 1).

Among patients with CTD-ILD overall, most were female (76.3% Optum^®^ EHR and 77.5% MarketScan) (Table 1). The median (interquartile range) age of CTD-ILD patients was 64 (55–72) years (Optum^®^ EHR) and 56 (48–62) years (MarketScan). In Optum^®^ EHR, RA-ILD patients were on average the oldest (median 67 years), and IIM-ILD and MCTD-ILD patients were the youngest (median 58 years). While the majority of the CTD-ILD Optum^®^ EHR population was Caucasian (72.1%), a higher proportion of patients with Black African ancestry had SLE-ILD (30.3%), IIM-ILD (24.5%), and MCTD (32.3%) relative to the other CTD-ILD subtypes (14.0%–17.1%).

**Table 1:** Baseline characteristics of CTD-ILD cohort

	Optum^®^ EHR							MarketScan						
	CTD-ILD overall^a^	RA-ILD	SSc-ILD	SLE-ILD	IIM-ILD	MCTD-ILD	SjD-ILD	CTD-ILD overall^a^	RA-ILD	SSc-ILD	SLE-ILD	IIM-ILD	MCTD-ILD	SjD-ILD
Number of patients, n (%)	21,366 (100.0)	10,278 (48.1)	4570 (21.4)	4033 (18.9)	2124 (9.9)	1966 (9.2)	4223 (19.8)	7521 (100.0)	3560 (47.3)	1678 (22.3)	1654 (22.0)	1103 (14.7)	810 (10.8)	1435 (19.1)
Age, years														
Mean (SD)	62.5 (12.8)	65.0 (11.6)	59.7 (12.9)	57.4 (14.1)	56.5 (13.8)	56.2 (13.8)	63.2 (12.3)	55.4 (12.5)	57.5 (12.2)	53.5 (11.6)	51.7 (12.4)	51.0 (11.9)	49.8 (12.0)	56.2 (12.3)
Median (IQR)	64 (55–72)	67 (58–74)	61 (52–69)	59 (48–68)	58 (48–67)	58 (47–67)	65 (56–72)	56 (48–62)	57 (50–64)	55 (46–60)	53 (43–59)	52 (43–59)	50 (43–57)	56 (49–63)
Age group, n (%)														
18–64 years	10,922 (51.1)	4440 (43.2)	2796 (61.2)	2664 (66.1)	1467 (69.1)	1371 (69.7)	2055 (48.7)	6118 (81.4)	2748 (77.2)	1469 (87.5)	1459 (88.2)	1020 (92.5)	746 (92.1)	1136 (79.2)
≥ 65 years	10,444 (48.9)	5838 (56.8)	1774 (38.8)	1369 (33.9)	657 (30.9)	595 (30.3)	2168 (51.3)	1403 (18.7)	812 (22.8)	209 (12.5)	195 (11.8)	83 (7.5)	64 (7.9)	299 (20.8)
Sex, n (%)														
Female	16,292 (76.3)	7409 (72.1)	3771 (82.5)	3511 (87.1)	1498 (70.5)	1663 (84.6)	3592 (85.1)	5828 (77.5)	2644 (74.3)	1389 (82.8)	1453 (87.9)	771 (69.9)	679 (83.8)	1230 (85.7)
Male	5046 (23.6)	2857 (27.8)	792 (17.3)	518 (12.8)	622 (29.3)	300 (15.3)	623 (14.8)	1693 (22.5)	916 (25.7)	289 (17.2)	201 (12.2)	332 (30.1)	131 (16.2)	205 (14.3)
Missing	28 (0.1)	12 (0.1)	7 (0.2)	4 (0.1)	4 (0.2)	3 (0.2)	8 (0.2)	0	0	0	0	0	0	0
Race, n (%)														
Caucasian	15,407 (72.1)	7853 (76.4)	3269 (71.5)	2358 (58.5)	1338 (63.0)	1068 (54.3)	3109 (73.6)	NA	NA	NA	NA	NA	NA	NA
Black African ancestry	3733 (17.5)	1436 (14.0)	781 (17.1)	1221 (30.3)	521 (24.5)	635 (32.3)	658 (15.6)	NA	NA	NA	NA	NA	NA	NA
Asian	739 (3.5)	318 (3.1)	185 (4.1)	144 (3.6)	97 (4.6)	95 (4.8)	158 (3.7)	NA	NA	NA	NA	NA	NA	NA
Missing	1487 (7.0)	671 (6.5)	335 (7.3)	310 (7.7)	168 (7.9)	168 (8.6)	298 (7.1)	NA	NA	NA	NA	NA	NA	NA

^a^Overall captures any CTD-ILD (no duplicates). Patients may be captured in more than one CTD subtype but will contribute only once to the CTD-ILD overall total

*CTD*, connective tissue disease; *EHR*, electronic health record; *IIM*, idiopathic inflammatory myopathy; *ILD*, interstitial lung disease; *IQR*, interquartile range; *MCTD*, mixed connective tissue disease; *NA*, not available; *RA*, rheumatoid arthritis; *SD*, standard deviation; *SjD*, Sjögren’s disease; *SLE*, systemic lupus erythematosus; *SSc*, systemic sclerosis

### Primary analysis: incidence

A total of 11,097 incident CTD-ILD cases were identified in Optum^®^ EHR, and 1925 in MarketScan during the study period. Crude incidence rates of CTD-ILD per 100,000 PY were reported as 8.0 [95% CI: 7.8, 8.1] in Optum^®^ EHR and 6.4 [95% CI: 6.2, 6.7] in MarketScan (Table 2). After age- and sex-adjustment, incidence rates per 100,000 PY were similar between the databases (7.6 [95% CI: 7.3, 7.9] for Optum^®^ EHR and 10.0 [95% CI: 9.3, 10.7] for MarketScan) (Table 2). Age and sex-adjusted incidence rates varied between CTD subtypes, and were highest for RA-ILD (3.9 [95% CI: 3.7, 4.1], Optum^®^ EHR, and 5.3 [95% CI: 4.8, 5.9], MarketScan) and lowest for MCTD-ILD (0.6 [95% CI: 0.6, 0.7], Optum^®^ EHR, and 0.6 [95% CI: 0.5, 0.8], MarketScan) (Table 2).

**Table 2:** Crude and adjusted incidence (per 100,000 PY) of CTD-ILD

	Optum^®^ EHR							MarketScan						
	CTD-ILD overall^a^	RA-ILD	SSc-ILD	SLE-ILD	IIM-ILD	MCTD-ILD	SjD-ILD	CTD-ILD overall^a^	RA-ILD	SSc-ILD	SLE-ILD	IIM-ILD	MCTD-ILD	SjD-ILD
Number of patients, n (%)	11,097 (100.0)	5646 (50.9)	2079 (18.7)	2054 (18.5)	952 (8.6)	922 (8.3)	2,325 (21.0)	1925 (100.0)	992 (51.5)	365 (19.0)	472 (24.5)	283 (14.7)	210 (10.9)	431 (22.4)
Adjusted incidence,^b^ (95% CI)	7.6 (7.3, 7.9)	3.9 (3.7, 4.1)	1.4 (1.3, 1.5)	1.4 (1.3, 1.5)	0.7 (0.6, 0.7)	0.6 (0.6, 0.7)	1.6 (1.4, 1.7)	10.0 (9.3, 10.7)	5.3 (4.8, 5.9)	1.7 (1.4, 2.0)	1.8 (1.6, 2.1)	1.3 (1.0, 1.5)	0.6 (0.5, 0.8)	2.3 (2.0, 2.7)
Crude incidence (95% CI)	8.0 (7.8, 8.1)	4.1 (4.0, 4.2)	1.5 (1.4, 1.6)	1.5 (1.4, 1.5)	0.7 (0.6, 0.7)	0.7 (0.6, 0.7)	1.7 (1.6, 1.7)	6.4 (6.2, 6.7)	3.3 (3.1, 3.5)	1.2 (1.1, 1.4)	1.6 (1.4, 1.7)	0.9 (0.8, 1.1)	0.7 (0.6, 0.8)	1.4 (1.3, 1.6)
Female	10.6 (10.4, 10.8)	5.1 (4.9, 5.2)	2.2 (2.1, 2.3)	2.2 (2.1, 2.4)	0.9 (0.8, 0.9)	1.0 (0.9, 1.0)	2.5 (2.4, 2.6)	9.8 (9.3, 10.3)	5.0 (4.6, 5.3)	2.0 (1.8, 2.2)	2.7 (2.4, 2.9)	1.3 (1.1, 1.5)	1.2 (1.0, 1.4)	2.4 (2.2, 2.7)
Male	4.4 (4.2, 4.6)	2.7 (2.5, 2.8)	0.6 (0.5, 0.7)	0.4 (0.4, 0.5)	0.4 (0.4, 0.5)	0.3 (0.2, 0.3)	0.5 (0.5, 0.6)	2.8 (2.6, 3.1)	1.6 (1.4, 1.8)	0.4 (0.3, 0.5)	0.4 (0.3, 0.6)	0.6 (0.5, 0.7)	0.2 (0.1, 0.3)	0.4 (0.3, 0.5)
Age 18–64 years	5.5 (5.3, 5.6)	2.4 (2.3, 2.5)	1.2 (1.2, 1.3)	1.3 (1.2, 1.4)	0.6 (0.6, 0.7)	0.6 (0.6, 0.7)	1.1 (1.0, 1.2)	6.0 (5.8, 6.3)	3.1 (2.9, 3.3)	1.2 (1.1, 1.3)	1.6 (1.4, 1.7)	0.9 (0.8, 1.0)	0.7 (0.6, 0.8)	1.3 (1.2, 1.5)
Age ≥ 65 years	16.4 (16.0, 16.9)	9.5 (9.1, 9.8)	2.4 (2.3, 2.6)	2.1 (1.9, 2.2)	0.9 (0.8, 1.0)	0.8 (0.7, 0.9)	3.6 (3.4, 3.8)	23.8 (20.3, 27.8)	13.1 (10.6, 16.2)	3.5 (2.4, 5.3)	2.8 (1.8, 4.4)	2.5 (1.5, 4.0)	0.5 (0.2, 1.4)	5.9 (4.3, 8.0)
Caucasian	7.7 (7.5, 7.9)	4.1 (4, 4.2)	1.4 (1.3, 1.5)	1.2 (1.1, 1.2)	0.6 (0.5, 0.6)	0.5 (0.5, 0.6)	1.6 (1.6, 1.7)	NA	NA	NA	NA	NA	NA	NA
Black African ancestry	11.0 (10.6, 11.6)	4.5 (4.2, 4.9)	2.0 (1.8, 2.3)	3.6 (3.3, 3.9)	1.4 (1.2, 1.6)	1.6 (1.4, 1.8)	2.0 (1.8, 2.3)	NA	NA	NA	NA	NA	NA	NA
Asian	8.7 (7.9, 9.6)	3.9 (3.4, 4.6)	2.2 (1.8, 2.6)	1.6 (1.2, 2.0)	1.0 (0.7, 1.3)	1.2 (0.9, 1.5)	1.9 (1.5, 2.3)	NA	NA	NA	NA	NA	NA	NA

^a^Overall captures any CTD-ILD (no duplicates). Patients may be captured in more than one CTD subtype but will contribute only once to the CTD-ILD overall total; ^b^Adjusted for age and sex

*CI*, confidence interval; *CTD*, connective tissue disease; *EHR*, electronic health record; *IIM*, idiopathic inflammatory myopathy; *ILD*, interstitial lung disease; *MCTD*, mixed connective tissue disease; *NA*, not available; *PY*, person-years; *RA*, rheumatoid arthritis; *SjD*, Sjögren’s disease; *SLE*, systemic lupus erythematosus; *SSc*, systemic sclerosis

The crude incidence rates for CTD-ILD overall were at least twice as high in female vs male patients (10.6 [95% CI: 10.4, 10.8] vs 4.4 [95% CI: 4.2, 4.6] in Optum^®^ EHR, and 9.8 [95% CI: 9.3, 10.3] vs 2.8 [95% CI: 2.6, 3.1] in MarketScan) (Table 2). This higher incidence in female vs male patients is consistent across all the CTD-ILD subtypes in both databases, with a female-to-male ratio ranging from 1.9 to 6.7 (Table 2). Overall crude incidence rates for CTD-ILD were also ≥ 3 times higher in patients aged ≥ 65 years than patients aged 18–64 years (16.4 [95% CI: 16.0, 16.9] vs 5.5 [95% CI: 5.3, 5.6] in Optum^®^ EHR, and 23.8 [95% CI: 20.3, 27.8] vs 6.0 [95% CI: 5.8, 6.3] in MarketScan) (Table 2). Higher incidence in older age groups was mostly consistent across CTD-ILD subtypes except for MCTD-ILD in MarketScan for which the estimate was lower in the older group, though confidence intervals were wide and overlapping. This is likely influenced by the age distribution in the MarketScan database (Table 2). Overall crude incidence rates were also slightly higher in individuals of Black African ancestry (11.0 [95% CI: 10.6, 11.6]), compared with Asian (8.7 [95% CI: 7.9, 9.6]) or Caucasian (7.7 [95% CI: 7.5, 7.9]) race in Optum^®^ EHR (Table 2); this was generally consistent across CTD-ILD subtypes. Crude incidence rates were consistently highest for RA-ILD across sex and age strata in both databases and across race strata in Optum EHR, whereas the lowest rates generally occurred for IIM-ILD or MCTD-ILD. A sensitivity analysis applying a 2-year washout period reported similar incidence rates for CTD-ILD (data not shown).

### Primary analysis: prevalence

The overall crude prevalence rate of CTD-ILD per 100,000 persons was higher in Optum^®^ EHR (47.1 [95% CI: 46.5, 47.7]) than in MarketScan (22.1 [95% CI: 21.6, 22.6]). After age- and sex-adjustment, prevalence rates were similar between the Optum^®^ EHR population (46.7 [95% CI: 45.6, 47.8]) and the MarketScan population (43.4 [95% CI: 42.2, 44.5]) (Table 3). Age- and sex-adjusted prevalence rates varied between CTD subtypes and were highest for RA-ILD (22.8 [95% CI: 22.0, 23.5] in Optum^®^ EHR, and 23.4 [95% CI: 22.5, 24.2] in MarketScan) and lowest for MCTD-ILD (4.2 [95% CI: 3.8, 4.5] in Optum^®^ EHR, and 3.1 [95% CI: 2.8, 3.4] in MarketScan) (Table 3).

**Table 3:** Crude and adjusted prevalence (per 100,000 persons) of CTD-ILD

	Optum^®^ EHR							MarketScan						
	CTD-ILD overall^a^	RA-ILD	SSc-ILD	SLE-ILD	IIM-ILD	MCTD-ILD	SjD-ILD	CTD-ILD overall^a^	RA-ILD	SSc-ILD	SLE-ILD	IIM-ILD	MCTD-ILD	SjD-ILD
Number of patients, n (%)	21,366 (100.0)	10,278 (48.1)	4570 (21.4)	4033 (18.9)	2124 (9.9)	1966 (9.2)	4223 (19.8)	7521 (100.0)	3560 (47.3)	1678 (22.3)	1654 (22.0)	1103 (14.7)	810 (10.8)	1435 (19.1)
Adjusted prevalence,^b^ (95% CI)	46.7 (45.6, 47.8)	22.8 (22.0, 23.5)	9.8 (9.3, 10.3)	8.6 (8.1, 9.0)	4.6 (4.3, 5.0)	4.2 (3.8, 4.5)	9.2 (8.7, 9.7)	43.4 (42.2, 44.5)	23.4 (22.5, 24.2)	7.7 (7.2, 8.2)	7.4 (6.9, 7.8)	4.1 (3.8, 4.5)	3.1 (2.8, 3.4)	8.7 (8.2, 9.3)
Crude prevalence (95% CI)	47.1 (46.5, 47.7)	22.7 (22.2, 23.1)	10.1 (9.8, 10.4)	8.9 (8.6, 9.2)	4.7 (4.5, 4.9)	4.3 (4.1, 4.5)	9.3 (9.0, 9.6)	22.1 (21.6, 22.6)	10.5 (10.1, 10.8)	4.9 (4.7, 5.2)	4.9 (4.6, 5.1)	3.2 (3.1, 3.4)	2.4 (2.2, 2.6)	4.2 (4.0, 4.4)
Female	64.6 (63.6, 65.6)	29.4 (28.7, 30.1)	15.0 (14.5, 15.4)	13.9 (13.5, 14.4)	5.9 (5.6, 6.2)	6.6 (6.3, 6.9)	14.3 (13.8, 14.7)	33.2 (32.3, 34.0)	15.1 (14.5, 15.6)	7.9 (7.5, 8.3)	8.3 (7.9, 8.7)	4.4 (4.1, 4.7)	3.9 (3.6, 4.2)	7.0 (6.6, 7.4)
Male	25.1 (24.4, 25.8)	14.2 (13.7, 14.7)	3.9 (3.7, 4.2)	2.6 (2.4, 2.8)	3.1 (2.9, 3.3)	1.5 (1.3, 1.7)	3.1 (2.9, 3.3)	10.3 (9.8, 10.8)	5.6 (5.2, 5.9)	1.8 (1.6, 2.0)	1.2 (1.1, 1.4)	2.0 (1.8, 2.2)	0.8 (0.7, 0.9)	1.3 (1.1, 1.4)
Age 18–64 years	30.6 (30.0, 31.2)	12.4 (12.1, 12.8)	7.8 (7.5, 8.1)	7.5 (7.2, 7.7)	4.1 (3.9, 4.3)	3.8 (3.6, 4.0)	5.8 (5.5, 6)	18.6 (18.1, 19.0)	8.3 (8.0, 8.7)	4.5 (4.2, 4.7)	4.4 (4.2, 4.7)	3.1 (2.9, 3.3)	2.3 (2.1, 2.4)	3.5 (3.3, 3.7)
Age ≥ 65 years	108.0 (105.9, 110.1)	60.4 (58.8, 61.9)	18.3 (17.5, 19.2)	14.2 (13.4, 14.9)	6.8 (6.3, 7.3)	6.2 (5.7, 6.7)	22.4 (21.5, 23.4)	132.6 (125.6, 139.5)	76.7 (71.4, 82.0)	19.8 (17.1, 22.4)	18.4 (15.8, 21.0)	7.8 (6.2, 9.5)	6.1 (4.6, 7.5)	28.3 (25.1, 31.5)
Caucasian	47.3 (46.6, 48.1)	24.1 (23.6, 24.7)	10.0 (9.7, 10.4)	7.2 (7.0, 7.5)	4.1 (3.9, 4.3)	3.3 (3.1, 3.5)	9.6 (9.2, 9.9)	NA	NA	NA	NA	NA	NA	NA
Black African ancestry	70.3 (68.1, 72.6)	27.1 (25.7, 28.5)	14.7 (13.7, 15.7)	23.0 (21.7, 24.3)	9.8 (9.0, 10.7)	12.0 (11.0, 12.9)	12.4 (11.5, 13.3)	NA	NA	NA	NA	NA	NA	NA
Asian	47.1 (43.7, 50.5)	20.3 (18.0, 22.5)	11.8 (10.1, 13.5)	9.2 (7.7, 10.7)	6.2 (5.0, 7.4)	6.1 (4.8, 7.3)	10.1 (8.5, 11.6)	NA	NA	NA	NA	NA	NA	NA

^a^Overall captures any CTD-ILD (no duplicates). Patients may be captured in more than one CTD subtype but will contribute only once to the CTD-ILD overall total; ^b^Adjusted for age and sex

*CI*, confidence interval; *CTD*, connective tissue disease; *EHR*, electronic health record; *IIM*, idiopathic inflammatory myopathy; *ILD*, interstitial lung disease; *MCTD*, mixed connective tissue disease; *NA*, not available; *RA*, rheumatoid arthritis; *SjD*, Sjögren’s disease; *SLE*, systemic lupus erythematosus; *SSc*, systemic sclerosis

Crude prevalence was consistently higher in female vs male patients for CTD-ILD overall and across subtypes (CTD-ILD overall: 64.6 [95% CI: 63.6, 65.6] vs 25.1 [95% CI: 24.4, 25.8] in Optum^®^ EHR, and 33.2 [95% CI: 32.3, 34.0] vs 10.3 [95% CI: 9.8, 10.8] in MarketScan); this was consistent across subtypes, with a female-to-male ratio of 1.9–6.8 (Table 3). The crude prevalence rate for CTD-ILD overall was at least 3.5 times higher in patients ≥ 65 years of age (relative to patients 18 to 64 years of age; 108.0 [95% CI: 105.9, 110.1] vs 30.6 [95% CI: 30.0, 31.2] in Optum^®^ EHR, and 132.6 [95% CI: 125.6, 139.5] vs 18.6 [95% CI: 18.1, 19.0] in MarketScan) (Table 3). This higher prevalence in older age groups was consistent across all the CTD-ILD subtypes with an older-to-younger ratio ranging between 1.7 and 9.2.

In the Optum^®^ EHR database, overall CTD-ILD crude prevalence rate was approximately 1.5 times higher in patients of Black African ancestry (70.3 [95% CI: 68.1, 72.6]) relative to Caucasian (47.3 [95% CI: 46.6, 48.1]) or Asian (47.1 [95% CI: 43.7, 50.5]) races; this varied across CTD-ILD subtypes, with the largest relative differences observed for SLE-ILD, IIM-ILD and MCTD-ILD (Table 3). Crude prevalence rates were also consistently highest for RA-ILD across the reported demographic strata, with the lowest estimates generally observed for IIM-ILD or MCTD-ILD.

### Sensitivity analysis: broad ILD definition

The sensitivity analysis using the broader ILD definition showed similar trends; however, age- and sex-adjusted incidence rates per 100,000 PYs for CTD-ILD overall were modestly increased compared with the primary definition in both databases (8.3 [95% CI: 8.0, 8.6] vs 7.6 [95% CI: 7.3, 7.9] for Optum^®^ EHR, and 10.8 [95% CI: 10.0, 11.5] vs 10.0 [95% CI: 9.3, 10.7] for MarketScan) (Supplementary Table S2 and Table 2). Age- and sex-adjusted prevalence rates for CTD-ILD overall were also slightly higher than those seen with the primary definition (52.7 [95% CI: 51.5, 53.8] vs 46.7 [95% CI: 45.6, 47.8] for Optum^®^ EHR, and 48.7 [95% CI: 47.5, 49.9] vs 43.4 [95% CI: 42.2, 44.5] for MarketScan) (Supplementary Table S3 and Table 3). Incidence and prevalence estimate increases were observed across most subtypes and subgroups, with findings similar to the primary analysis.

### Sensitivity analysis: 365-day enrollment

Age- and sex-adjusted incidence rates for CTD-ILD overall were similar when using 365-day enrollment criteria (7.6 [95% CI: 7.4, 7.9] Optum^®^ EHR, and 10.1 [95% CI: 9.4, 10.9] MarketScan) (Supplementary Table S4) compared to the primary analysis that used a 1-day enrollment criteria (Table 2). The age- and sex-adjusted prevalence rates for CTD-ILD overall were higher when using the 365-day enrollment criteria (54.2 [95% CI: 52.9, 55.5] Optum^®^ EHR, and 52.8 [95% CI: 51.3, 54.4] MarketScan) (Supplementary Table S5) compared with those seen with the primary definition (Table 3). For both incidence and prevalence, relative patterns across subtypes and subgroups remain unchanged versus the primary analysis.

### Sensitivity analysis: multi-CTD-ILD separated

Across both databases, separating patients with multiple CTD diagnoses into a distinct group resulted in lower incidence and prevalence estimates for all CTD-ILD subtypes (Supplementary Table S6). Age- and sex-adjusted incidence estimates decreased by 22%–80% across individual CTD-ILD subtypes, while adjusted prevalence estimates decreased by 23%–70%, with the greatest reductions observed for MCTD-ILD, SLE-ILD, and SjD-ILD. Despite these reductions, the relative pattern across CTD subtypes remained broadly consistent, with RA-ILD remaining the most common and MCTD-ILD the least common subtype. The age- and sex-adjusted incidence rates for the multi-CTD-ILD group were 1.5 and 2.3 per 100,000 person-years in Optum^®^ EHR and MarketScan, respectively; prevalence rates were 9.6 and 8.6 per 100,000 persons. The multi-CTD-ILD subgroup represented approximately 20%–31% of incident and prevalent cases across the databases. Relative patterns across subtypes and subgroups were similar to the primary analyses. These findings indicate that overlapping CTD diagnoses contribute substantially to subtype-specific estimates but do not materially alter the overall epidemiological patterns observed in the primary analysis.

## Discussion

This large retrospective cohort study estimated the age- and sex-adjusted incidence rates of CTD-ILD overall at 7.6 (Optum^®^ EHR) and 10.0 (MarketScan) per 100,000 PY, and prevalence rates at 46.7 (Optum^®^ EHR) and 43.4 (MarketScan) per 100,000 persons between 2019 and 2024, in a US healthcare-utilizing population. CTD-ILD overall crude incidence and prevalence rates were at least double in female vs male patients and at least 3 times higher in patients ≥ 65 years of age compared with younger age groups across both databases. In Optum^®^ EHR, crude incidence and prevalence rates of CTD-ILD overall were higher in patients of Black African ancestry compared with Caucasian or Asian patients, indicating potential racial disparities in CTD-ILD risk.

After adjusting for differences in age structure of the databases, the results across two large US databases (Optum^®^ EHR and MarketScan) provided similar age- and sex-adjusted incidence and prevalence rates of CTD-ILD overall and by CTD subtype. Age- and sex-adjusted incidence and prevalences rates varied between CTD subtypes and were consistently highest for RA-ILD and lowest in MCTD-ILD across both databases. Although incidence and prevalence were consistently higher in female vs male patients and higher in older age groups across all CTD-ILD subtypes, the magnitude of the difference varied across subtypes. These novel data provide insights into the rate of CTD-ILD across different populations.

Existing published evidence of CTD-ILD incidence and prevalence in the US, overall and by individual subtypes, is limited, with data available predominantly for RA-ILD and SSc-ILD. Within RA-ILD, a prior study in the US using MarketScan reported crude annual incidence and prevalence rates between 2004 and 2013 that were lower than reported here [14]. This study reported outcomes only annually, had an older study period that used ICD-9 diagnoses codes, and used a different case definition, all of which could contribute to these lower rates. For SSc-ILD, two published studies included incidence and prevalence estimates; both studies used administrative claims databases in the US but used slightly different algorithms and ICD-10 codes to define ILD than the current study [21, 22]. However, for SSc-ILD, the definitions that were most similar to our algorithm, explored in a sensitivity analysis (i.e. required two SSc codes and two ILD codes), yielded similar results to our study [21].

The use of a validated algorithm to consistently identify ILD across all the six CTD subtypes addresses an important gap in the literature and strengthens estimates of CTD-ILD prevalence and incidence, which have typically been assessed separately using heterogenous methodology. The prevalence of CTD-ILDs is often reported as the proportion of patients with CTD who also have ILD. This study reports proportions between 2.7% (RA-ILD) and 21.1% (SSc-ILD). These proportions have varied substantially among other studies, as reported by several systematic reviews and meta-analyses [12, 24, 25]. A study by Gupta et al., including a variety of study types, reported proportions of each CTD with ILD as 0.6%–63.7% (RA-ILD), 26.1%–88.1% (SSc-ILD), 1%–87.8% (SjD-ILD), 27.5%–61.3% (IIM-ILD) and 40% (MCTD-ILD) [24]. The variation demonstrated in the literature is likely due to many factors, such as the population source used in the study, study sample size, healthcare system, and the definition and identification of CTD-ILD. Studies using ICD codes commonly report proportions much lower than those relying on high-resolution computer tomography to diagnose CTD-ILD. Delays in diagnosis of CTD-ILD, which have been frequently reported as several months or years, are also a likely factor for the observed variation in reports [26].

Demographic characteristics examined in this study, such as female sex, older age, and Black African ancestry, were found to consistently impact rates of CTD-ILD incidence and prevalence. Although some of these factors have been previously identified as risk factors for individual CTD-ILDs, e.g. older age (RA-ILD, SLE-ILD, and SSc-ILD) and Black African ancestry (SSc-ILD) [9, 20], this study only examines the risk of CTD-ILD overall, which can be driven by the risk of the underlying CTD. For example, while incidence and prevalence of RA-ILD in females is higher than males, this is likely due to the higher frequency of RA occurrence in females. Whereas, amongst patients with RA, males have been found to have a higher risk of developing ILD than females [9]. While identifying risk factors for developing ILD was not the focus of this study, it represents an important area for future investigation. The findings from this study help the understanding of the epidemiology and burden of CTD-ILD. The heterogeneity in CTD-ILD frequency across subtypes has implications for healthcare planning, resource allocation, and the development of disease specific care pathways and can help guide the development of targeted therapies and strategies aimed at improving patient care and outcomes.

### Strengths and limitations

This study benefits from the use of two large and complementary population-based databases. The Optum^®^ EHR database was selected because of its large population size, enabling robust evaluation of uncommon CTD-ILD subtypes. However, electronic health record data are collected primarily for routine clinical care and primarily represent individuals who engage with participating healthcare networks. The analysis was replicated in the MarketScan claims database, which captures healthcare encounters recorded for reimbursement among individuals with employer-sponsored insurance. Because the two databases represent different US populations and have distinct strengths and limitations, their combined use provided broader population coverage and enabled validation of findings across distinct real-world data sources. The consistency of age- and sex-adjusted incidence and prevalence estimates across both databases supports the robustness of the estimates, particularly for the less common CTD-ILD subtypes. Despite the wide coverage of the US population, these results may not be generalizable to the whole US CTD-ILD population, particularly those without commercial insurance. Therefore, age- and sex-adjusted rates were calculated to improve the generalizability of the findings and to help mitigate potential biases related to demographic differences in the databases.

As with all studies using administrative healthcare data, diagnoses were not clinically validated, and case identification relied on coding algorithms, some of which are unvalidated. Additionally, the lack of more CTD-ILD subtype-specific supportive criteria may limit accuracy across different subtypes. Although the ILD algorithm used in this study was previously validated in US patients with RA [23], with a similar code set used in a validation study in IIM-ILD using Hospital Episode Statistics data from the United Kingdom [27], validated subtype-specific CTD-ILD algorithms are not currently available. Consequently, algorithm performance may differ across CTD subtypes and data sources. In addition, healthcare provider specialty, chest CT findings and pulmonary function testing were not consistently available across both databases and therefore not incorporated into the case definition. Sensitivity analyses using broader ILD definitions and alternative enrollment and washout criteria found only a modest increase in estimates, supporting the robustness of the primary analysis in capturing ILD across CTD subtypes. Nevertheless, future validation studies are required to establish validated subtype-specific CTD-ILD identification algorithms. Another limitation in our study is the occurrence of multiple CTD diagnoses in some patients, which may result in a person being captured in more than one CTD-ILD subtype. Multiple recorded diagnoses may reflect true overlapping syndromes, evolution of the underlying autoimmune diagnosis over time, or limitations in diagnostic coding. The sensitivity analysis demonstrated that separation of these patients into a distinct multi-CTD-ILD group reduced the subtype-specific incidence and prevalence estimates, although the overall patterns remained largely unchanged. These results highlight an important methodological challenge that is rarely addressed in published studies and should be considered when comparing subtype-specific estimates across studies.

### Conclusions

This study provides robust and novel estimates of the incidence and prevalence of CTD-ILD in the US, drawing on data from two large and demographically distinct databases. The findings highlight that older age, female sex, and Black African ancestry are associated with higher observed rates of CTD-ILD. By improving our understanding of the epidemiology of CTD-ILD, this research offers insights that can guide the development of targeted therapies and inform strategies to enhance disease screening and management, patient care, and healthcare resource allocation.

## Supplementary Information

Below is the link to the electronic supplementary material.

Supplementary file1 (DOCX 78.3 KB)

## Data Availability

For requests for access to anonymized subject level data, please contact the corresponding author.

## Declarations

### Previous presentation

Part of this research was previously presented at the European Alliance of Associations for Rheumatology (EULAR) Congress held in Barcelona, Spain (June 11–14, 2025), published in the *Annals of the Rheumatic Diseases* (Martins D, et al. *Ann Rheum Dis*. 2025;84(1):1408–9) and the American College of Rheumatology (ACR) 2025 Annual Meeting held in Chicago, USA (October 24–29, 2025), and is published in *Arthritis & Rheumatology* (Martins D, et al. *Arthritis Rheumatol.* 2025; 77 (suppl 9)).
