# Supplementary material for: Incidence and prevalence of connective tissue disease (CTD)-associated interstitial lung disease in the United States
Source: Clin Rheumatol. 2026 Oct 23;45(12):7147–57. doi: 10.1007/s10067-026-08480-6 (PMC13755075; doi:10.1007/s10067-026-08480-6)
Supplement: Supplementary file 1 [file 10067_2026_8480_MOESM1_ESM.docx]

## **Supplementary materials**

## **Supplementary Table S1. Identification of CTD-ILD cohorts**

| **Primary and broad sensitivity definitions** | | |
| --- | --- | --- |
| **Cohort** | **ICD-10 codes** | **Claims** |
| RA [1] | M05.X, M06.X | ≥2 IP or OP claims ≥7 days and ≤365 days apart and ≥1 prescription for DMARD use |
| SLE [2, 3] | M32.X except M32.0 | ≥2 OP claims >30 days and ≤365 days apart |
| IIM [4, 5] | M33.X, M36.0, M60.8X, G72.4 | ≥1 IP or ≥2 OP claims ≥30 days and ≤365 days apart |
| SjD [6] | M35.0X | ≥1 IP or ≥2 OP claims ≥7 days and ≤365 days apart |
| MCTD | M35.1 | ≥1 IP or ≥2 OP claims ≥7 days and ≤365 days apart |
| SSc [7] | M34.X | ≥2 IP or OP claims ≥7 days and ≤365 days apart |
| ILD [8]  (Primary definition) | J84.1X, J84.89, J84.9 | ≥2 IP or OP claims ≥30 days apart |
| ILD (broad definition)^a^ | J84.1X, J84.89, J84.9  M05.1X (Rheumatoid lung disease with RA),  M32.13 (Lung involvement in SLE),  M34.81 (SSc with lung involvement),  M35.02 (SjD with lung involvement),  M33.11 (Other dermatomyositis with respiratory involvement),  M33.21 (Polymyositis with respiratory involvement),  M33.91 (Dermatopolymyositis, unspecified with respiratory involvement) |  |

CTD diagnosis date: date fulfilling each algorithm; ILD diagnosis date: first date fulfilling algorithm; CTD-ILD index date: latest of CTD and ILD date that meets inclusion criteria.

CTD, connective tissue disease; DMARD, disease‑modifying antirheumatic drug; ICD-10, International Classification of Diseases 10th Revision; IIM, idiopathic inflammatory myopathy; ILD, interstitial lung disease; IP, inpatient; MCTD, mixed connective tissue disease; OP, outpatient; PPV, positive predictive value; RA, rheumatoid arthritis; SjD, Sjögren’s disease; SLE, systemic lupus erythematosus; SSc, systemic sclerosis.

## **Supplementary Table S2. Crude and adjusted incidence (per 100,000 PY) of CTD-ILD (sensitivity analysis broader definition)**

|  | **Optum**® **EHR** | | | | | | | **MarketScan** | | | | | | | |
| --- | --- | --- | --- | --- | --- | --- | --- | --- | --- | --- | --- | --- | --- | --- | --- |
|  | **CTD-ILD overall^a^** | **RA-ILD** | **SSc-ILD** | **SLE-ILD** | **IIM-ILD** | **MCTD-ILD** | **SjD-ILD** | **CTD-ILD  overall^a^** | **RA-ILD** | **SSc-ILD** | **SLE-ILD** | **IIM-ILD** | **MCTD-ILD** | **SjD-ILD** |  |
| **Number of patients, n (%)** | 12,194 (100.0) | 5914  (48.5) | 2203  (18.1) | 2526  (20.7) | 1030  (8.4) | 922  (7.6) | 2514  (20.6) | 2175  (100.0) | 1065  (49.0) | 377  (17.3) | 564  (25.9) | 309  (14.2) | 212  (9.7) | 485  (22.3) |  |
| **Adjusted incidence,^b^ (95% CI)** | 8.3  (8.0, 8.6) | 4.1  (3.9, 4.3) | 1.5  (1.4, 1.6) | 1.7  (1.6, 1.8) | 0.7  (0.6, 0.8) | 0.6  (0.6, 0.7) | 1.7  (1.6, 1.8) | 10.8  (10.0, 11.5) | 5.5  (4.9, 6.0) | 1.7  (1.4, 2.0) | 2.1  (1.8, 2.3) | 1.5  (1.2, 1.7) | 0.7  (0.5, 0.8) | 2.6  (2.2, 2.9) |  |
| **Crude incidence (95% CI)** | 8.8  (8.6, 8.9) | 4.3  (4.2, 4.4) | 1.6  (1.5, 1.7) | 1.8  (1.8, 1.9) | 0.7  (0.7, 0.8) | 0.7  (0.6, 0.7) | 1.8  (1.7, 1.9) | 7.3  (7.0, 7.6) | 3.6  (3.4, 3.8) | 1.3  (1.1, 1.4) | 1.9  (1.7, 2.0) | 1.0  (0.9, 1.2) | 0.7  (0.6, 0.8) | 1.6  (1.5, 1.8) |  |
| Female | 11.7  (11.5, 12.0) | 5.3  (5.2, 5.5) | 2.3  (2.2, 2.4) | 2.8  (2.6, 2.9) | 0.9  (0.9, 1.0) | 1.0  (0.9, 1.0) | 2.7  (2.6, 2.8) | 11.1  (10.6, 11.7) | 5.3  (5.0, 5.7) | 2.0  (1.8, 2.3) | 3.2  (2.9, 3.5) | 1.4  (1.2, 1.6) | 1.2  (1.0, 1.4) | 2.7  (2.5, 3) |  |
| Male | 4.7  (4.5, 4.9) | 2.8  (2.7, 2.9) | 0.6  (0.5, 0.7) | 0.5  (0.5, 0.6) | 0.5  (0.4, 0.5) | 0.3  (0.2, 0.3) | 0.6  (0.5, 0.6) | 3.2  (2.9, 3.5) | 1.7  (1.5, 1.9) | 0.4  (0.3, 0.6) | 0.5  (0.4, 0.6) | 0.7  (0.6, 0.8) | 0.2  (0.1, 0.3) | 0.4  (0.3, 0.6) |  |
| Age 18–64 years | 6.1  (6.0, 6.3) | 2.6  (2.5, 2.7) | 1.3  (1.2, 1.4) | 1.7  (1.6, 1.7) | 0.7  (0.6, 0.7) | 0.6  (0.6, 0.7) | 1.2  (1.1, 1.3) | 6.9  (6.6, 7.2) | 3.3  (3.1, 3.6) | 1.2  (1.1, 1.3) | 1.9  (1.7, 2.0) | 1.0  (0.9, 1.1) | 0.7  (0.6, 0.8) | 1.5  (1.4, 1.7) |  |
| Age ≥65 years | 17.5  (17.1, 18.0) | 9.9  (9.5, 10.2) | 2.6  (2.4, 2.8) | 2.3  (2.2, 2.5) | 1  (0.9, 1.1) | 0.8  (0.7, 0.9) | 3.9  (3.7, 4.1) | 24.8  (21.3, 29.0) | 13.3  (10.7, 16.4) | 3.5  (2.4, 5.3) | 2.8  (1.8, 4.4) | 3.1  (2.0, 4.8) | 0.5  (0.2, 1.4) | 6.2  (4.5, 8.4) |  |
| Caucasian | 8.4  (8.3, 8.6) | 4.3  (4.2, 4.5) | 1.5  (1.4, 1.6) | 1.4  (1.4, 1.5) | 0.6  (0.6, 0.7) | 0.5  (0.5, 0.6) | 1.8  (1.7, 1.9) | NA | NA | NA | NA | NA | NA | NA |  |
| Black African ancestry | 12.2  (11.7, 12.7) | 4.7  (4.4, 5.0) | 2.1  (1.9, 2.3) | 4.3  (4.0, 4.6) | 1.4  (1.3, 1.6) | 1.6  (1.4, 1.8) | 2.2  (2.0, 2.4) | NA | NA | NA | NA | NA | NA | NA |  |
| Asian | 9.6  (8.7, 10.5) | 4.0  (3.4, 4.6) | 2.3  (1.9, 2.7) | 2.1  (1.7, 2.6) | 1.0  (0.8, 1.4) | 1.2  (0.9, 1.5) | 2.0  (1.6, 2.5) | NA | NA | NA | NA | NA | NA | NA |  |

^a^Overall captures any CTD-ILD (no duplicates). Patients may be captured in more than one CTD subtype but will contribute only once to the CTD-ILD overall total; ^b^Adjusted for age and sex.
CI; confidence interval; CTD, connective tissue disease; EHR, electronic health record; IIM, idiopathic inflammatory myopathy; ILD, interstitial lung disease; MCTD, mixed connective tissue disease; NA, not available; PY, person-years; RA, rheumatoid arthritis; SjD, Sjögren’s disease; SLE, systemic lupus erythematosus; SSc, systemic sclerosis.

## **Supplementary Table S3. Crude and adjusted prevalence (per 100,000 persons) of CTD-ILD (sensitivity analysis broader definition)**

|  | **Optum**® **EHR** | | | | | | | **MarketScan** | | | | | | | |
| --- | --- | --- | --- | --- | --- | --- | --- | --- | --- | --- | --- | --- | --- | --- | --- |
|  | **CTD-ILD overall^a^** | **RA-ILD** | **SSc-ILD** | **SLE-ILD** | **IIM-ILD** | **MCTD-ILD** | **SjD-ILD** | | **CTD-ILD  overall^a^** | **RA-ILD** | **SSc-ILD** | **SLE-ILD** | **IIM-ILD** | **MCTD-ILD** | **SjD-ILD** |
| **Number of patients, n (%)** | 24,146  (100.0) | 2321  (9.6) | 1966  (8.1) | 11,008  (45.6) | 5132  (21.3) | 4928  (20.4) | 4685  (19.4) | | 8762  (100.0) | 3916  (44.7) | 2124  (24.2) | 2124  (24.2) | 1215  (13.9) | 812  (9.3) | 1622  (18.5) |
| **Adjusted prevalence,^b^ (95% CI)** | 52.7  (51.5, 53.8) | 24.4  (23.6, 25.2) | 10.6  (10.1, 11.1) | 10.8  (10.3, 11.4) | 5.0  (4.7, 5.4) | 4.2  (3.8, 4.5) | 10.2  (9.7, 10.7) | | 48.7  (47.5, 49.9) | 25.3  (24.5, 26.2) | 8.3  (7.8, 8.8) | 8.7  (8.2, 9.3) | 4.6  (4.3, 5.0) | 3.1  (2.8, 3.4) | 9.8  (9.2, 10.3) |
| **Crude prevalence (95% CI)** | 53.2  (52.5, 53.9) | 24.3  (23.8, 24.7) | 10.9  (10.6, 11.2) | 11.3  (11, 11.6) | 5.1  (4.9, 5.3) | 4.3  (4.1, 4.5) | 10.3  (10.0, 10.6) | | 25.8  (25.2, 26.3) | 11.5  (11.2, 11.9) | 5.4  (5.1, 5.6) | 6.3  (6.0, 6.5) | 3.6  (3.4, 3.8) | 2.4  (2.2, 2.6) | 4.8  (4.5, 5.0) |
| Female | 73.8  (72.8, 74.9) | 31.5  (30.8, 32.2) | 16.2  (15.7, 16.7) | 17.8  (17.2, 18.3) | 6.5  (6.2, 6.8) | 6.6  (6.3, 6.9) | 15.9  (15.4, 16.4) | | 39.0  (38.1, 40.0) | 16.6  (16.0, 17.2) | 8.6  (8.2, 9.0) | 10.7  (10.2, 11.2) | 4.8  (4.5, 5.1) | 3.9  (3.6, 4.2) | 8.0  (7.6, 8.4) |
| Male | 27.4  (26.7, 28.2) | 15.2  (14.7, 15.7) | 4.1  (3.8, 4.4) | 3.2  (3.0, 3.5) | 3.4  (3.1, 3.7) | 1.5  (1.3, 1.7) | 3.3  (3.1, 3.6) | | 11.6  (11.1, 12.1) | 6.1  (5.7, 6.5) | 1.9  (1.7, 2.1) | 1.5  (1.3, 1.7) | 2.3  (2.1, 2.5) | 0.8  (0.7, 0.9) | 1.3  (1.2, 1.5) |
| Age 18–64 years | 35.6  (35.0, 36.2) | 13.4  (13.0, 13.8) | 8.4  (8.1, 8.7) | 9.9  (9.6, 10.3) | 4.5  (4.3, 4.7) | 3.8  (3.6, 4.0) | 6.5  (6.2, 6.8) | | 21.9  (21.4, 22.5) | 9.2  (8.9, 9.6) | 4.9  (4.6, 5.1) | 5.8  (5.6, 6.1) | 3.4  (3.2, 3.6) | 2.3  (2.1, 2.4) | 3.9  (3.7, 4.1) |
| Age ≥65 years | 118.3  (116.1, 120.4) | 64.4  (62.8, 66.0) | 19.8  (18.9, 20.7) | 16.4  (15.6, 17.2) | 7.5  (6.9, 8.0) | 6.2  (5.7, 6.7) | 24.5  (23.5, 25.5) | | 145.1  (137.9, 152.4) | 82.5  (77.0, 88.0) | 21.2  (18.4, 23.9) | 19.8  (17.2, 22.5) | 9.1  (7.3, 10.9) | 6.1  (4.6, 7.5) | 31.3  (27.9, 34.6) |
| Caucasian | 53.4  (52.6, 54.2) | 25.9  (25.4, 26.5) | 10.9  (10.5, 11.2) | 9.3  (9.0, 9.6) | 4.5  (4.3, 4.8) | 3.3  (3.1, 3.5) | 10.7  (10.3, 11.1) | | NA | NA | NA | NA | NA | NA | NA |
| Black African ancestry | 79.9  (77.5, 82.3) | 28.6  (27.2, 30.0) | 15.7  (14.6, 16.7) | 28.6  (27.2, 30.1) | 10.5  (9.6, 11.3) | 12.0  (11.0, 12.9) | 13.5  (12.5, 14.5) | | NA | NA | NA | NA | NA | NA | NA |
| Asian | 52.6  (49, 56.2) | 21.4  (19.1, 23.6) | 12.5  (10.8, 14.2) | 11.7  (10.0, 13.4) | 6.8  (5.5, 8.1) | 6.1  (4.8, 7.3) | 10.8  (9.2, 12.5) | | NA | NA | NA | NA | NA | NA | NA |

^a^Overall captures any CTD-ILD (no duplicates). Patients may be captured in more than one CTD subtype but will contribute only once to the CTD-ILD overall total; ^b^Adjusted for age and sex.
CI, confidence interval; CTD, connective tissue disease; EHR, electronic health record; IIM, idiopathic inflammatory myopathy; ILD, interstitial lung disease; MCTD, mixed connective tissue disease; NA, not available; RA, rheumatoid arthritis; SjD, Sjögren’s disease; SLE, systemic lupus erythematosus; SSc, systemic sclerosis.

## **Supplementary Table S4. Crude and adjusted incidence (per 100,000 PY) of CTD-ILD (sensitivity analysis 365-day enrollment^a^)**

|  | **Optum**® **EHR** | | | | | | | | **MarketScan** | | | | | | |
| --- | --- | --- | --- | --- | --- | --- | --- | --- | --- | --- | --- | --- | --- | --- | --- |
|  | **CTD-ILD overall^b^** | **RA-ILD** | **SSc-ILD** | **SLE-ILD** | **IIM-ILD** | **MCTD-ILD** | **SjD-ILD** | **CTD-ILD  overall^b^** | | **RA-ILD** | **SSc-ILD** | **SLE-ILD** | **IIM-ILD** | **MCTD-ILD** | **SjD-ILD** |
| **Number of patients, n (%)** | 11,012  (100.0) | 5607  (50.9) | 2062  (18.7) | 2035  (18.5) | 947  (8.6) | 919  (8.3) | 2312  (21.0) | 1900  (100.0) | | 983  (51.7) | 362  (19.1) | 467  (24.6) | 278  (14.6) | 206  (10.8) | 421  (22.2) |
| **Adjusted incidence,^c^ (95% CI)** | 7.6  (7.4, 7.9) | 3.9  (3.7, 4.1) | 1.4  (1.3, 1.5) | 1.4  (1.3, 1.5) | 0.7  (0.6, 0.7) | 0.6  (0.6, 0.7) | 1.6  (1.5, 1.7) | 10.1  (9.4, 10.9) | | 5.4  (4.9, 6.0) | 1.7  (1.5, 2.0) | 1.8  (1.6, 2.1) | 1.2  (1.0, 1.5) | 0.7  (0.5, 0.8) | 2.3  (2.0, 2.7) |
| **Crude incidence (95% CI)** | 8.1  (7.9, 8.2) | 4.1  (4.0, 4.2) | 1.5  (1.4, 1.6) | 1.5  (1.4, 1.6) | 0.7  (0.7, 0.7) | 0.7  (0.6, 0.7) | 1.7  (1.6, 1.8) | 6.5  (6.2, 6.8) | | 3.4  (3.2, 3.6) | 1.2  (1.1, 1.4) | 1.6  (1.5, 1.8) | 1.0  (0.9, 1.1) | 0.7  (0.6, 0.8) | 1.4  (1.3, 1.6) |
| Female | 10.7  (10.4, 10.9) | 5.1  (5.0, 5.3) | 2.2  (2.1, 2.3) | 2.3  (2.2, 2.4) | 0.9  (0.8, 0.9) | 1.0  (0.9, 1.0) | 2.5  (2.4, 2.6) | 9.9  (9.4, 10.4) | | 5.1  (4.7, 5.4) | 2.0  (1.8, 2.3) | 2.7  (2.4, 3.0) | 1.3  (1.1, 1.5) | 1.2  (1.0, 1.4) | 2.4  (2.2, 2.7) |
| Male | 4.4  (4.3, 4.6) | 2.7  (2.6, 2.8) | 0.6  (0.5, 0.7) | 0.4  (0.4, 0.5) | 0.5  (0.4, 0.5) | 0.3  (0.2, 0.3) | 0.5  (0.5, 0.6) | 2.9  (2.6, 3.2) | | 1.6  (1.4, 1.8) | 0.4  (0.3, 0.5) | 0.4  (0.3, 0.6) | 0.6  (0.5, 0.8) | 0.2  (0.1, 0.3) | 0.4  (0.3, 0.5) |
| Age 18–64 years | 5.5  (5.4, 5.7) | 2.5  (2.4, 2.6) | 1.2  (1.2, 1.3) | 1.3  (1.2, 1.4) | 0.6  (0.6, 0.7) | 0.6  (0.6, 0.7) | 1.1  (1.0, 1.2) | 6.1  (5.9, 6.4) | | 3.2  (3.0, 3.4) | 1.2  (1.1, 1.3) | 1.6  (1.4, 1.7) | 0.9  (0.8, 1.0) | 0.7  (0.6, 0.8) | 1.4  (1.2, 1.5) |
| Age ≥65 years | 16.5  (16.0, 16.9) | 9.5  (9.2, 9.9) | 2.5  (2.3, 2.6) | 2.1  (1.9, 2.3) | 0.9  (0.8, 1.0) | 0.8  (0.7, 0.9) | 3.6  (3.4, 3.8) | 23.9  (20.4, 28.1) | | 13.2  (10.7, 16.4) | 3.6  (2.4, 5.4) | 2.8  (1.8, 4.5) | 2.4  (1.4, 3.9) | 0.5  (0.2, 1.5) | 5.8  (4.2, 8.0) |
| Caucasian | 7.8  (7.6, 7.9) | 4.2  (4.0, 4.3) | 1.4  (1.4, 1.5) | 1.2  (1.1, 1.2) | 0.6  (0.5, 0.6) | 0.5  (0.5, 0.6) | 1.7  (1.6, 1.7) | NA | | NA | NA | NA | NA | NA | NA |
| Black African ancestry | 11.0  (10.5, 11.6) | 4.5  (4.2, 4.9) | 2.0  (1.8, 2.3) | 3.6  (3.3, 3.9) | 1.4  (1.2, 1.6) | 1.6  (1.4, 1.8) | 2.1  (1.9, 2.3) | NA | | NA | NA | NA | NA | NA | NA |
| Asian | 8.8  (8.0, 9.7) | 3.9  (3.4, 4.6) | 2.2  (1.8, 2.7) | 1.6  (1.3, 2.0) | 1.0  (0.8, 1.4) | 1.2  (0.9, 1.5) | 1.9  (1.5, 2.4) | NA | | NA | NA | NA | NA | NA | NA |

^a^Primary ILD definition with 365-day enrollment criteria; ^b^Overall captures any CTD-ILD (no duplicates). Patients may be captured in more than one CTD subtype but will contribute only once to the CTD-ILD overall total; ^c^Adjusted for age and sex.
CI, confidence interval; CTD, connective tissue disease; EHR, electronic health record; IIM, idiopathic inflammatory myopathy; ILD, interstitial lung disease; MCTD, mixed connective tissue disorder; NA, not available; RA, rheumatoid arthritis; SjD, Sjögren’s disease; SLE, systemic lupus erythematosus; SSc, systemic sclerosis.

## **Supplementary Table S5. Crude and adjusted prevalence (per 100,000 persons) of CTD-ILD (sensitivity analysis 365-day enrollment^a^)**

|  | **Optum**® **EHR** | | | | | | | | **MarketScan** | | | | | | |
| --- | --- | --- | --- | --- | --- | --- | --- | --- | --- | --- | --- | --- | --- | --- | --- |
|  | **CTD-ILD  overall^b^** | **RA-ILD** | **SSc-ILD** | **SLE-ILD** | **IIM-ILD** | **MCTD-ILD** | **SjD-ILD** | **CTD-ILD overall^b^** | | **RA-ILD** | **SSc-ILD** | **SLE-ILD** | **IIM-ILD** | **MCTD-ILD** | **SjD-ILD** |
| **Number of patients, n (%)** | 20,617  (100.0) | 10,012  (48.6) | 4359  (21.1) | 3892  (18.9) | 2041  (9.9) | 1917  (9.3) | 4079  (19.8) | 7123  (100.0) | | 3386  (47.5) | 1584  (22.2) | 1572  (22.1) | 1042  (14.6) | 769  (10.8) | 1362  (19.1) |
| **Adjusted prevalence,^c^ (95% CI)** | 54.2  (52.9, 55.5) | 26.6  (25.7, 27.5) | 11.3  (10.7, 11.9) | 9.9  (9.4, 10.5) | 5.4  (4.9, 5.8) | 4.9  (4.5, 5.3) | 10.6  (10.0, 11.2) | 52.8  (51.3, 54.4) | | 28.1  (27.0, 29.2) | 9.6  (9.0, 10.3) | 9.4  (8.8, 10.1) | 5.4  (4.9, 5.8) | 4.0  (3.6, 4.4) | 10.5  (9.8, 11.2) |
| **Crude prevalence (95% CI)** | 55.5  (54.8, 56.3) | 27.0  (26.4, 27.5) | 11.7  (11.4, 12.1) | 10.5  (10.2, 10.8) | 5.5  (5.3, 5.7) | 5.2  (4.9, 5.4) | 11.0  (10.7, 11.3) | 31.8  (31.1, 32.6) | | 15.1  (14.6, 15.6) | 7.1  (6.7, 7.4) | 7.0  (6.7, 7.4) | 4.7  (4.4, 4.9) | 3.4  (3.2, 3.7) | 6.1  (5.8, 6.4) |
| Female | 75.2  (74.0, 76.4) | 34.5  (33.7, 35.3) | 17.3  (16.7, 17.8) | 16.2  (15.6, 16.7) | 6.9  (6.5, 7.2) | 7.8  (7.4, 8.1) | 16.7  (16.1, 17.2) | 47.6  (46.4, 48.9) | | 21.8  (21.0, 22.6) | 11.3  (10.7, 11.9) | 11.9  (11.3, 12.5) | 6.3  (5.8, 6.7) | 5.6  (5.1, 6.0) | 10.1  (9.5, 10.7) |
| Male | 29.9  (29.1, 30.8) | 17.2  (16.6, 17.8) | 4.6  (4.2, 4.9) | 3.1  (2.8, 3.4) | 3.7  (3.4, 4.0) | 1.8  (1.6, 2.0) | 3.6  (3.3, 3.9) | 14.8  (14.1, 15.5) | | 7.9  (7.4, 8.5) | 2.5  (2.2, 2.8) | 1.8  (1.5, 2.0) | 2.9  (2.6, 3.3) | 1.2  (1.0, 1.4) | 1.7  (1.5, 2.0) |
| Age 18–64 years | 36.6  (35.9, 37.3) | 15.1  (14.6, 15.5) | 9.3  (8.9, 9.6) | 8.9  (8.5, 9.2) | 4.9  (4.7, 5.2) | 4.6  (4.4, 4.9) | 6.9  (6.6, 7.2) | 26.9  (26.2, 27.6) | | 12.1  (11.7, 12.6) | 6.4  (6.1, 6.8) | 6.4  (6.1, 6.8) | 4.5  (4.2, 4.8) | 3.3  (3.1, 3.5) | 5.0  (4.7, 5.3) |
| Age ≥65 years | 123.0  (120.6, 125.4) | 69.4  (67.5, 71.2) | 20.6  (19.6, 21.6) | 16.2  (15.3, 17.0) | 7.6  (7.0, 8.2) | 7.2  (6.6, 7.7) | 25.5  (24.4, 26.6) | 147.4  (139.6, 155.3) | | 85.1  (79.1, 91.1) | 21.9  (18.9, 24.9) | 20.9  (18.0, 23.9) | 8.6  (6.7, 10.5) | 6.8  (5.1, 8.4) | 31.0  (27.4, 34.6) |
| Caucasian | 54.0  (53.2, 54.9) | 27.7  (27.1, 28.3) | 11.4  (11.0, 11.8) | 8.3  (7.9, 8.6) | 4.7  (4.4, 4.9) | 3.8  (3.6, 4.1) | 10.9  (10.5, 11.3) | NA | | NA | NA | NA | NA | NA | NA |
| Black African ancestry | 81.8  (79.1, 84.4) | 32.1  (30.4, 33.7) | 16.9  (15.6, 18.1) | 26.7  (25.2, 28.3) | 11.4  (10.4, 12.4) | 13.9  (12.8, 15.0) | 14.5  (13.4, 15.6) | NA | | NA | NA | NA | NA | NA | NA |
| Asian | 57.0  (52.9, 61.2) | 24.8  (22.1, 27.6) | 13.9  (11.9, 16) | 11.3  (9.4, 13.1) | 7.6  (6.1, 9.1) | 7.5  (5.9, 9.0) | 12.4  (10.4, 14.3) | NA | | NA | NA | NA | NA | NA | NA |

^a^Primary definition with 365-day enrollment criteria; ^b^Overall captures any CTD-ILD (no duplicates). Patients may be captured in more than one CTD subtype but will contribute only once to the CTD-ILD overall total; ^c^Adjusted for age and sex.
CI, confidence interval; CTD, connective tissue disease; EHR, electronic health record; IIM, idiopathic inflammatory myopathy; ILD, interstitial lung disease; MCTD, mixed connective tissue disease; NA, not available; PY, person-years; RA, rheumatoid arthritis; SjD, Sjögren’s disease; SLE, systemic lupus erythematosus; SSc, systemic sclerosis.

## **Supplementary Table S6: Adjusted incidence (per 100,000 PY) and prevalence (per 100,000 persons) for CTD-ILD (sensitivity analysis separating patients with multiple CTD diagnoses^a^)**

|  | **Optum**® **EHR** | | | | | | | **MarketScan** | | | | | | |
| --- | --- | --- | --- | --- | --- | --- | --- | --- | --- | --- | --- | --- | --- | --- |
|  | **RA-ILD** | **SSc-ILD** | **SLE-ILD** | **IIM-ILD** | **MCTD-ILD** | **SjD-ILD** | **Multi-CTD-ILD** | **RA-ILD** | **SSc-ILD** | **SLE-ILD** | **IIM-ILD** | **MCTD-ILD** | **SjD-ILD** | **Multi-CTD-ILD** |
| **Incidence** |  |  |  |  |  |  |  |  |  |  |  |  |  |  |
| **Number of patients, n (%)** | 4374 (36.9) | 1379 (13.9) | 1027 (9.2) | 581 (6.1) | 308 (2.8) | 1195 (9.8) | 2269 (100.0) | 611 (45.9) | 194 (14.6) | 186 (14.0) | 143 (10.7) | 37 (2.8) | 161 (12.1) | 606 (100.0) |
| **Adjusted incidence,^a^**  **(95% CI)** | 3.0 (2.9, 3.2) | 0.9 (0.8, 1.0) | 0.7 (0.6, 0.8) | 0.4 (0.4, 0.5) | 0.2 (0.2, 0.3) | 0.8 (0.7, 0.9) | 1.5 (1.4, 1.6) | 4.0 (3.5, 4.6) | 1.0 (0.8, 1.3) | 0.8 (0.6, 1.0) | 0.8 (0.6, 1.1) | 0.1 (0.1, 0.2) | 1.0 (0.8, 1.3) | 2.3 (2.0, 2.6) |
| **Crude incidence (95% CI)** | 3.2  (3.1, 3.3) | 1.0  (0.9, 1.1) | 0.7  (0.7, 0.8) | 0.4  (0.4, 0.5) | 0.2  (0.2, 0.3) | 0.9  (0.8, 0.9) | 1.6  (1.6, 1.7) | 2.0  (1.9, 2.2) | 0.7  (0.6, 0.8) | 0.6  (0.5, 0.7) | 0.5  (0.4, 0.6) | 0.1  (0.1, 0.2) | 0.5  (0.5, 0.6) | 2.0  (1.9, 2.2) |
| Female | 3.7  (3.6, 3.8) | 1.4  (1.3, 1.5) | 1.1  (1.0, 1.2) | 0.5  (0.4, 0.5) | 0.3  (0.3, 0.3) | 1.2  (1.1, 1.3) | 2.5  (2.4, 2.6) | 2.8  (2.6, 3.1) | 1.1  (0.9, 1.2) | 1.0  (0.8, 1.1) | 0.6  (0.5, 0.7) | 0.2  (0.2, 0.3) | 0.9  (0.8, 1.1) | 3.4  (3.1, 3.7) |
| Male | 2.4  (2.3, 2.5) | 0.5  (0.4, 0.5) | 0.3  (0.2, 0.3) | 0.4  (0.3, 0.4) | 0.1  (0.1, 0.2) | 0.4  (0.3, 0.4) | 0.4  (0.4, 0.5) | 1.2  (1.1, 1.4) | 0.2  (0.2, 0.3) | 0.3  (0.2, 0.4) | 0.4  (0.3, 0.4) | 0.0  (0.0, 0.1) | 0.2  (0.1, 0.4) | 0.6  (0.5, 0.7) |
| Age 18–64 years | 1.7  (1.6, 1.8) | 0.8  (0.7, 0.8) | 0.6  (0.6, 0.7) | 0.4  (0.3, 0.4) | 0.2  (0.2, 0.2) | 0.5  (0.4, 0.5) | 1.1  (1.3, 1.5) | 1.8  (1.7, 2.0) | 0.6  (0.5, 0.7) | 0.6  (0.5, 0.7) | 0.4  (0.4, 0.5) | 0.1  (0.1, 0.2) | 0.5  (0.4, 0.6) | 2.0  (1.8, 2.2) |
| Age ≥65 years | 8.0  (7.7, 8.3) | 1.8  (1.6, 1.9) | 1.2  (1.1, 1.3) | 0.6  (0.5, 0.7) | 0.4  (0.3, 0.4) | 2.1  (2.0, 2.3) | 3.6  (2.3, 2.6) | 11.6  (9.2, 14.5) | 2.5  (1.5, 4.0) | 1.5  (0.8, 2.9) | 2.2  (1.3, 3.7) | 0.2  (0.0, 1.1) | 2.8  (1.8, 4.4) | 3.4  (2.2, 5.2) |
| Caucasian | 3.3  (3.2, 3.4) | 1.0  (0.9, 1.0) | 0.6  (0.6, 0.6) | 0.4  (0.3, 0.4) | 0.2  (0.2, 0.2) | 0.9  (0.8, 0.9) | 1.4  (1.3, 1.5) | NA | NA | NA | NA | NA | NA | NA |
| Black African ancestry | 2.9  (2.7, 3.2) | 1.2  (1.0, 1.4) | 1.8  (1.6, 2.0) | 0.8  (0.6, 0.9) | 0.4  (0.3, 0.5) | 0.9  (0.8, 1.0) | 3.2  (2.9, 3.5) | NA | NA | NA | NA | NA | NA | NA |
| Asian | 2.8  (2.4, 3.3) | 1.3  (1.0, 1.7) | 0.8  (0.6, 1.1) | 0.7  (0.5, 1.0) | 0.3  (0.2, 0.6) | 1.0  (0.7, 1.3) | 2.0  (1.7, 2.5) | NA | NA | NA | NA | NA | NA | NA |
| **Prevalence** |  |  |  |  |  |  |  |  |  |  |  |  |  |  |
| **Number of patients, n (%)** | 7811 (46.9) | 2942 (17.7) | 1944 (11.7) | 1292 (7.8) | 600 (3.6) | 2079 (12.5) | 4523 (100.0) | 2340 (43.2) | 993 (18.3) | 716 (13.2) | 581 (10.7) | 202 (3.7) | 583 (10.7) | 2039 (100.0) |
| **Adjusted prevalence,^a^**  **(95% CI)** | 17.5 (16.8, 18.2) | 6.4 (6.0, 6.8) | 4.2 (3.8, 4.5) | 2.8 (2.6, 3.1) | 1.3 (1.1, 1.5) | 4.6 (4.3, 4.9) | 9.6 (9.1, 10.1) | 18.1 (17.4, 18.9) | 5.0 (4.6, 5.4) | 3.7 (3.4, 4.0) | 2.4 (2.2, 2.7) | 0.9 (0.7, 1.1) | 4.3 (3.9, 4.6) | 8.6 (8.1, 9.1) |
| **Crude prevalence (95% CI)** | 17.2  (16.9, 17.6) | 6.5  (6.3, 6.7) | 4.3  (4.1, 4.5) | 2.9  (2.7, 3.0) | 1.3  (1.2, 1.4) | 4.6  (4.4, 4.8) | 10.0  (9.7, 10.3) | 6.9  (6.6, 7.2) | 2.9  (2.7, 3.1) | 2.1  (2.0, 2.3) | 1.7  (1.6, 1.9) | 0.6  (0.5, 0.7) | 1.7  (1.6, 1.9) | 6.0  (5.7, 6.3) |
| Female | 21.0  (20.4, 21.5) | 9.3  (9.0, 9.7) | 6.4  (6.1, 6.7) | 3.3  (3.0, 3.5) | 1.8  (1.7, 2.0) | 6.6  (6.3, 6.9) | 15.7  (15.2, 16.2) | 9.2  (8.7, 9.6) | 4.6  (4.3, 4.9) | 3.5  (3.2, 3.7) | 2.1  (1.9, 2.3) | 0.9  (0.8, 1.1) | 2.7  (2.5, 3.0) | 9.9  (9.5, 10.4) |
| Male | 12.6  (12.1, 13.1) | 2.9  (2.7, 3.2) | 1.6  (1.4, 1.8) | 2.3  (2.1, 2.6) | 0.7  (0.6, 0.8) | 2.1  (1.9, 2.3) | 2.7  (2.5, 3.0) | 4.5  (4.2, 4.8) | 1.1  (1.0, 1.3) | 0.7  (0.5, 0.8) | 1.3  (1.1, 1.5) | 0.3  (0.2, 0.3) | 0.6  (0.5, 0.8) | 1.8  (1.6, 2.0) |
| Age 18–64 years | 8.2  (7.9, 8.5) | 4.8  (4.6, 5.0) | 3.4  (3.2, 3.6) | 2.4  (2.2, 2.6) | 1.0  (0.9, 1.1) | 2.4  (2.2, 2.6) | 8.1  (7.8, 8.4) | 5.1  (4.8, 5.3) | 2.6  (2.4, 2.7) | 1.8  (1.7, 2.0) | 1.6  (1.5, 1.7) | 0.5  (0.5, 0.6) | 1.3  (1.2, 1.4) | 5.5  (5.3, 5.8) |
| Age ≥65 years | 50.6  (49.2, 52.0) | 12.7  (12.0, 13.4) | 7.7  (7.1, 8.2) | 4.5  (4.1, 5.0) | 2.5  (2.2, 2.8) | 12.7  (12.0, 13.4) | 16.8  (16.0, 17.6) | 64.1  (59.2, 68.9) | 14.1  (11.8, 16.4) | 10.4  (8.5, 12.4) | 5.5  (4.1, 6.9) | 2.2  (1.3, 3.1) | 15.2  (12.9, 17.6) | 20.3  (17.6, 23.0) |
| Caucasian | 19.2  (18.6, 19.6) | 6.7  (6.5, 7.0) | 3.7  (3.4, 3.9) | 2.6  (2.4, 2.8) | 1.2  (1.1, 1.3) | 4.9  (4.7, 5.1) | 8.8  (8.5, 9.1) | NA | NA | NA | NA | NA | NA | NA |
| Black African ancestry | 16.5  (15.4, 17.5) | 8.2  (7.4, 8.9) | 10.4  (9.5, 11.3) | 5.3  (4.7, 5.9) | 3.0  (2.5, 3.4) | 5.1  (4.5, 5.7) | 21.3  (20.1, 22.6) | NA | NA | NA | NA | NA | NA | NA |
| Asian | 14.6  (12.7, 16.4) | 7.4  (6.1, 8.8) | 4.2  (3.2, 5.2) | 3.6  (2.7, 4.6) | 1.3  (0.7, 1.8) | 4.3  (3.3, 5.4) | 11.5  (9.8, 13.2) | NA | NA | NA | NA | NA | NA | NA |

^a^Adjusted for age and sex.

CI, confidence interval; CTD, connective tissue disease; EHR, electronic health record; IIM, idiopathic inflammatory myopathy; ILD, interstitial lung disease; MCTD, mixed connective tissue disease; NA, not available; PY, person-years; RA, rheumatoid arthritis; SjD, Sjögren’s disease; SLE, systemic lupus erythematosus; SSc, systemic sclerosis.

**References**

1.	Kim SY, Servi A, Polinski JM, et al. (2011) Validation of rheumatoid arthritis diagnoses in health care utilization data. Arthritis Research & Therapy 13:R32. <https://doi.org/10.1186/ar3260>

2.	Li S, Gong T, Peng Y, Nieman KM, Gilbertson DT (2020) Prevalence and incidence of systemic lupus erythematosus and associated outcomes in the 2009-2016 US Medicare population. Lupus 29:15-26. <https://doi.org/10.1177/0961203319888691>

3.	Feldman C, Curtis JR, Yazdany J, Izmirly P, Oates JC (2024) Validating claims-based algorithms for a systemic lupus erythematosus diagnosis in Medicare data for informed use of the Lupus Index: a tool for geospatial research. Lupus Science & Medicine 11:e001329. <https://doi.org/10.1136/lupus-2024-001329>

4.	Furst DE, Amato AA, Iorga SR, Gajria K, Fernandes AW (2012) Epidemiology of adult idiopathic inflammatory myopathies in a U.S. managed care plan. Muscle Nerve 45:676-83. <https://doi.org/10.1002/mus.23302>

5.	Hannah JR, Gordon PA, Galloway J, et al. (2022) Validation of methods to identify people with idiopathic inflammatory myopathies using hospital episode statistics. Rheumatology Advances in Practice 6. <https://doi.org/10.1093/rap/rkac102>

6.	Birt J, Tan Y, Lin CY, Wang Y, Mozaffarian N (2015) AB0526 Patient Characteristics, Treatment Patterns, Resource Utilization and Costs of Sjogren's Syndrome Using A U.S. Managed Care Prescription Claims Database. Ann Rheum Dis 74:1076. <https://doi.org/10.1136/annrheumdis-2015-eular.1071>

7.	Fan Y, Bender S, Shi W, Zoz D (2020) Incidence and prevalence of systemic sclerosis and systemic sclerosis with interstitial lung disease in the United States. J Manag Care Spec Pharm 26:1539-47. <https://doi.org/10.18553/jmcp.2020.20136>

8.	England BR, Roul P, Mahajan TD, et al. (2020) Performance of Administrative Algorithms to Identify Interstitial Lung Disease in Rheumatoid Arthritis. Arthritis Care Res (Hoboken) 72:1392-403. <https://doi.org/10.1002/acr.24043>
